# Effect of labio-lingual inclination on mandibular anterior teeth displacement and stress distribution with fixed lingual retainers: A finite element study

**DOI:** 10.6026/973206300220947

**Published:** 2026-02-28

**Authors:** Gunjan Bhansali, Javed Sodawala, Mohammad Ullah Khan, Heli Dholakia, Amar Kingaonkar, Rajnee Yadav

**Affiliations:** 1 Department of Orthodontics and Dentofacial Orthopedics, VYWS Dental College and Hospital, Tapovan-Wadali Road, Amravati, Maharashtra, India; 2 Department of Orthodontics and Dentofacial Orthopedics, Chhattisgarh Dental College and Research Institute, Sundara, GE Road, Rajnandgaon, Chhattisgarh, India; 3 Department of Orthodontist, Tobal Dental Clinic, Buraidah, Al Qassim, Saudi Arabia; 4 Department of Orthodontist, Glen Orthodontics, Glenview, USA; 5 Department of Dentistry, BSP Medical College, Bhalgaon, Chhatrapati, Sambhajinagar, Maharashtra, India; 6 Department of Prosthodontics, Eklavya Dental college & Hospital, Kotputli, Jaipur, Rajasthan, India

**Keywords:** Labio-lingual displacement, von mises stresses, strain energy, mandibular anterior teeth, fixed lingual retainer

## Abstract

Labio-lingual displacement and stress distribution of mandibular anterior teeth may be altered by changes in incisor inclination, and the role of a lower lingual fixed retainer in modifying these effects remains unclear. Therefore, it is of interest to determine the effect of the presence of a lower lingual fixed retainer and variation in labio-lingual inclination on displacement patterns and cervico-apical stress distribution of surrounding tissues. Eight 3D finite element models of the anterior mandible were constructed, and incisor inclination was modified to 80°C, 90°C, 100°C, and 110°C relative to the mandibular plane in retainer and non- retainer groups. A vertical load of 187 N was applied to evaluate labio-lingual displacement, periodontal ligament (PDL) stress distribution, and strain energy. The findings showed that displacement patterns, cervico-apical stress distribution, and strain energy were comparable in both groups. It can be concluded that mandibular anterior inclination influences biomechanical response, while the presence of a lingual fixed retainer does not significantly alter stress distribution or displacement patterns.

## Background:

In the realm of orthodontics, the use of fixed retainers has become increasingly prevalent, particularly for stabilizing results in the mandibular arch [1, 2-3]. Initially, stainless steel round wires were the standard, but advancements in materials have led to the adoption of multistranded, braided and coaxial round wires, each offering varying compositions and resilience. Despite their widespread use, the precise effects of fixed retainers on tooth displacement and the distribution of occlusal forces on the periodontal tissues remain inadequately understood. This gap in knowledge is particularly critical as the post-treatment inclination of the mandibular anterior teeth can significantly differ based on whether patients undergo extraction or non-extraction treatments. Patients with extraction treatment plans tend to have more upright teeth, while those with non-extraction treatments may exhibit proclined mandibular anterior teeth. Understanding these differences is essential for optimizing retention strategies. Finite element method (FEM), a computational technique, has been widely employed to evaluate the stress distribution patterns on the mandibular anterior teeth and surrounding structures, both in healthy and periodontally compromised conditions. Gerami *et al.* [4] investigated the displacement and force distribution of splinted and tilted mandibular anterior teeth under occlusal loads using FEM. However, this study did not compare these findings with the normal displacement and force distribution patterns of non-splinted mandibular anterior teeth. Therefore, it is of interest to evaluate and compare the degree of displacement of mandibular anterior teeth, stress distribution patterns around supporting tissues and the strain energy in the mandibular anterior teeth at different labio-lingual inclinations {Incisor Mandibular Plane Angle (IMPA) = 80°C, 90°C, 100°C and 110°C} with and without the presence of a lingual fixed retainer.

## Methodology:

Cone beam computerized tomography (CBCT) scan of the anterior region of mandible with well-aligned teeth was recorded using Acteon X-MIND® PRIME 3D (Aceton India Pvt. Ltd, Gandhinagar, India) with a slice thickness of 1 mm. These CBCT scans were used to construct FEM models.

## Construction of FEM models:

**Figure 1:**
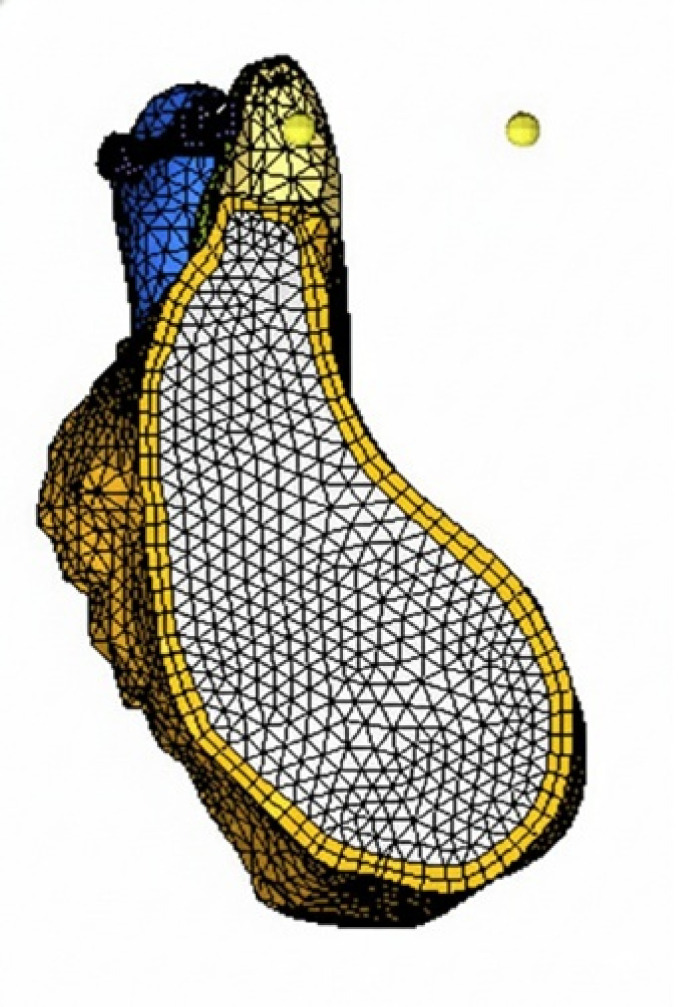
Cross-section of meshed models of experimental group (E) with incisor mandibular plane angles (IMPA) of 90°C

Eight 3-dimensional (3D) finite element models of mandibular anterior segment were constructed using HyperMesh® software (Version 11, Altair® Engineering, Inc, USA). The models included six anterior well-aligned teeth of mandible with average dimensions and supporting structures. Each model consisted of a cancellous bone surrounded by a 1 mm thick cortical bone. A uniform 0.25 mm thick periodontal ligament (PDL) was modeled based on the root form geometry of the teeth [3, 5]. These models were divided into small tetrahedral elements with 10 nodes, assuming that the teeth, alveolar bone, PDL and lingual retainer are homogenous elastic bodies. The models were divided into two groups based on the presence of bonded fixed lingual retainer. In control (C) group, 4 models were made with IMPA of 80°C, 90°C, 100°C and 110°C. (Model C80, C90, C100 and C110 respectively). In experimental (E) group, 4 models were made with a pentaflex lingual fixed retainer (78-LP00-0195, GC Orthodontics, TOMY Inc, Japan) bonded with composite tags (4 mm x 2 mm, Transbond XT, 3M Unitek, Monrovia, CA, USA) and IMPA 80°C, 90°C, 100°C and 110°C (Model E80, E90, E100 and E110 respectively) (Figure 1, Figure 2A, Figure 2B). The number of tetrahedral elements and nodes used in all the models are illustrated in Table 1. Figure 2A, Figure 2B meshed model of control and experimental groups with incisor mandibular plane angle (IMPA) of 90°C. Figure 3A, Figure 3B incisal and apical displacement of lower anterior teeth in experimental (E) and control (C) groups with incisor mandibular plane angles (IMPA) of 90°C.

## Processing of FEM models:

The models were transferred to the Ansys® software (Version 18.1, ANSYS Inc, Southpointe, Pittsburg, USA) for processing. Teeth supported by the PDL were represented by a tooth element. The relationship between teeth, their PDL, spongy and cortical bone and the pentaflex wire with composite and teeth was provided by contact elements. All vital tissues were presumed elastic, homogenous and isotropic. Compounding elastic properties such as Young's modulus and Poisson's ratio were applied Table 2. The pentaflex lingual fixed retainer was attached to the lingual surface of the mandibular anterior teeth by composite tags with an elastic beam element. A vertical force of 187 N was applied at each incisal edge of the central incisors [4]. All rigid body motions were prevented. The inclination of teeth in each model was indicated by markers on the occlusal and apical ends of the teeth. The boundary conditions were also defined to simulate how the models were constrained and to prevent them from free body motion. The nodes attached to the area of the outer surface of the bone were fixed in all directions to avoid free movements. Tooth displacement of mandibular anterior teeth in the incisal and apical direction, change of strain energy in the mandibular anterior teeth and von Mises stresses in the PDL in the cervical and apical parts were assessed and compared between the control group and the experimental group.

## Results:

In both control and experimental group, the central incisor showed the maximum incisal displacement in C80 and E80 models and the maximum apical displacement in C110 and E110 models. The maximum change in incisal displacement was observed in the central incisor between the C100 and E100 models in both groups. Similarly, the maximum change in apical displacement was observed in the central incisor between the C110 and E110 models in both groups. Figure 3A, Figure 3B, Table 3. The von mises stresses observed in the PDL of the anterior teeth in the experimental group were higher cervically and lower apically across all models. In the control group, the highest von Mises stresses were present cervically and apically on the lateral incisors in the C110 model. In the experimental group, the highest von Mises stresses were present on the central incisor cervically in the E80 model and apically in the E100 model. The maximum change in von Mises stresses was observed in the lateral incisor both cervically as well as apically between C110 and E110 models in both groups Table 4. The strain energy was highest in the C80 model in the control group, whereas it was highest in the E110 model in the experimental group Table 5.

## Discussion:

The viscoelastic nature of periodontal tissues, coupled with adaptations in anatomic characteristics such as bone mass, bone level and the width of the PDL space, is key to physiologic tooth mobility [6]. The wire in a fixed retainer can undergo elastic deflection by being mechanically deformed under masticatory loads [7]. Artun *et al.* [8, 9-10] found that the lingual fixed retainer did not cause any significant unwanted changes to the adjacent hard and soft tissues in the long run. Fixed retainers were thought to cause tooth displacement and increased periodontal stresses because of the masticatory forces acting on them. Many authors [4, 5, 11 and 12] studied the displacement and stresses produced on mandibular anterior teeth after placement of lingual fixed retainer but did not compare their results with a control group. Therefore, this study evaluated the displacement of teeth and stresses produced with and without a lingual fixed retainer on mandibular anterior teeth with different inclinations.

Sifakakis *et al.* [13] evaluated the vertical and labio-lingual forces generated by the fixed lingual retainer on mandibular anterior teeth and found that force levels upto 1 N can be produced if wire is displaced upto 0.2 mm, which can cause unwanted tooth movement during retention. The results observed in the present study shows teeth displacement <0.02 mm (incisally and apically) for both the control and the experimental groups, suggesting that the displacement was within the physiologic limit. In the present study, when forces were applied, the incisal and apical displacement was similar in models with and without fixed lingual retainers, except when mandibular incisors were proclined and IMPA was 100°C and 110°C. The von Misses stresses were also similar in both groups except for lateral incisors when mandibular incisors were proclined and IMPA was 110°C. The strain energy was slightly less when mandibular anterior teeth were bonded with the fixed lingual retainers. Gerami *et al.* [4] investigated the inclination of the mandibular anterior teeth and its association with dental displacement with a fixed lingual retainer, finding more stresses on the central and lateral incisors when mandibular incisors were more proclined. Similar results were seen in the experimental group in the present study. Jahanbin *et al.* [14] in his FEM study observed that different positions of Fiber Reinforced Composite (FRC) and Flexible Spiral Wire (FSW) retainers produced different amounts of displacement for the mandibular anterior teeth. For FRC retainer attached at middle third of the lingual surface, maximum displacement was found for central incisors. In the present study, pentaflex retainer was attached at the middle third of the lingual surface of mandibular anterior teeth and similar maximum displacement was observed for central incisors. Geramy *et al.* [5] found that the von Mises stresses produced in the PDL of the anterior teeth increased after splinting them with a fixed lingual retainer. However, the stresses observed in the present study were lesser than 0.005 MPa, in the both the control and experimental groups and do not pose a risk of any significant damage to the teeth and the surrounding periodontium.

Gerami *et al.* [4] observed that increasing the inclination of the mandibular anterior teeth also increased the strain energy produced on them. Similar results were seen in the present study, but the values of strain energy were comparatively less. Moradinejad *et al.* [15] showed flexible multistrand wire retainers superior to CAD/CAM NiTi in reducing PDL stresses and displacements under 187N loads in resorption cases, aligning with our finding of unchanged labio-lingual patterns and cervical stress distribution with pentaflex retainers across inclinations. Zhang *et al.* [16] found 0.5-mm stainless-steel retainers viable without resorption but with central stress risks under biting, consistent with our results of similar displacement and von Mises stresses in PDL with retainers versus controls. Kukreja *et al.* [17] demonstrated 3D-printed chrome-cobalt wires outperforming stainless-steel in load stability, complementing our evidence that flexible pentaflex retainers induce no undesirable strain energy changes under vertical forces. Zeng *et al.* [18] reported greater central incisor labial displacement in bone loss models, supporting our observation of incisal/apical central movements but confirming retainers maintain equivalent cervico-apical stress patterns to non-retained teeth. The differences in the values seen may be because of the difference in the methodologies. The models in our study have been fabricated by obtaining CBCT scan of mandibular anterior segment of a patient unlike previous studies [4, 10] where ideal values were used to fabricate models. Also, different software was used for modeling. The structural and spatial relationships of various dental components differ among individuals, contributing to varying responses and potentially differing clinical outcomes. In this study, the PDL was modeled as a layer of uniform thickness and treated as linear, elastic and isotropic, even though it exhibits anisotropic and non-linear viscoelastic behavior due its tissue fluids. There was a single point of force application. Future long-term clinical studies need to evaluate the changes in mandibular anterior teeth due to fixed lingual retainer under constant loading.

## Conclusion:

The experimental group bonded with a fixed lingual retainer on mandibular anterior teeth showed labio-lingual displacement patterns almost similar to those of control group. The models with different IMPA angles were evaluated and the displacement observed in all the models was physiologic. No unwanted changes were seen in the group splinted with fixed lingual retainer. The cervico-apical stress distribution pattern in the PDL of mandibular anterior teeth was found to be similar in both study groups. No harmful stresses were generated in the PDL after bonding the mandibular anterior teeth with a fixed lingual retainer. Additionally, the strain energy was similar in both study groups. Therefore, it can be concluded that the lingual fixed retainer does not cause any unwanted changes on the mandibular anterior teeth.

## Advancement to knowledge:

This FEM study advances orthodontic knowledge by demonstrating through 3D models that lower lingual fixed retainers do not cause undesirable labio-lingual displacements or changes in cervico-apical PDL stress distribution in mandibular anterior teeth across varying inclinations (80°C-110°C), with only minor strain energy increases. It addresses a research gap by isolating the retainer's effect, unlike prior studies on general PDL stresses. Findings support routine retainer use without heightened relapse or tissue remodeling risks.

## Figures and Tables

**Figure 2A:**
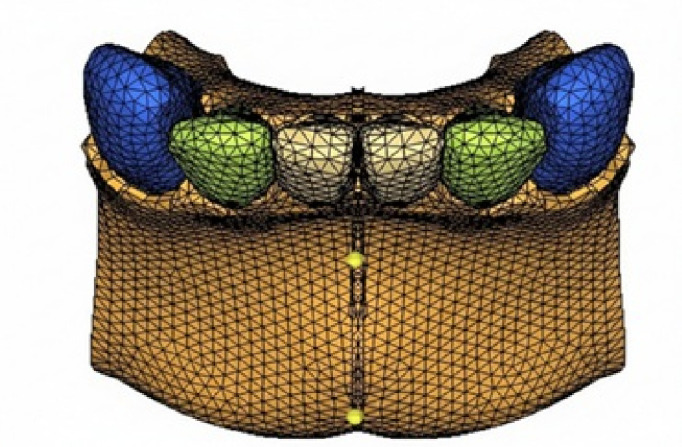
C90 model with IMPA of 90°C

**Figure 2B:**
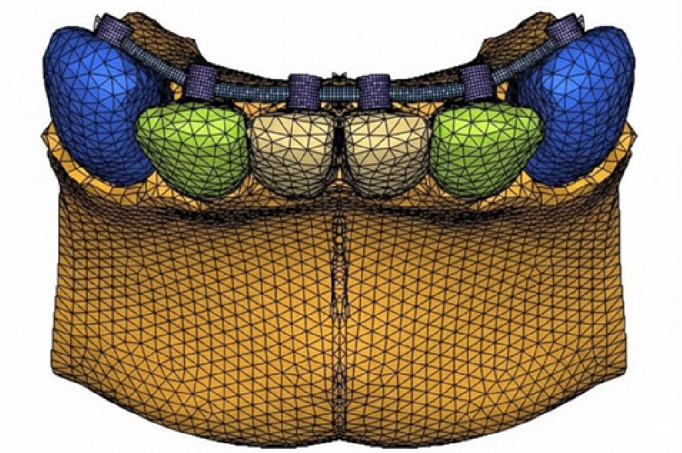
E90 model with IMPA of 90°C

**Figure 3A:**
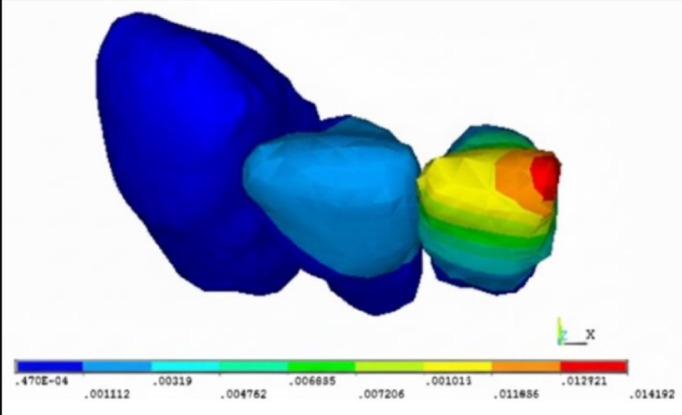
E90 model with IMPA of 90°C

**Figure 3B:**
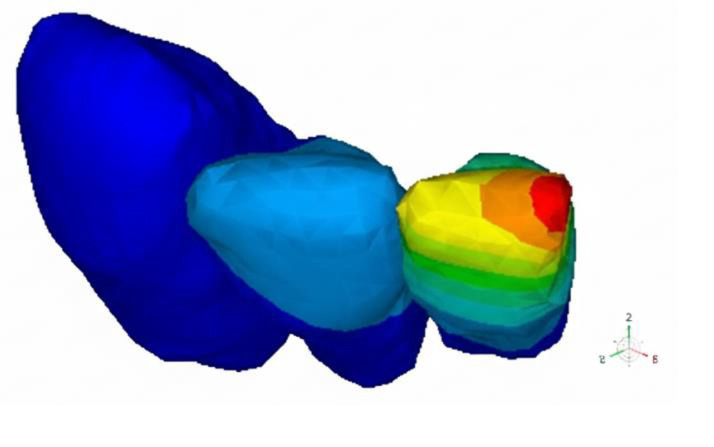
C90 model with IMPA of 90°C

**Table 1:** Number of elements and nodes of the models

**Models**	**Number of elements**	**Number of nodes**
Central incisor	2073	3678
Lateral incisor	2325	4055
Canine	3273	5563
Periodontal ligament (PDL)	1689	3452
Composite	8869	15801
Spongy bone	16096	26609
Cortical bone	8670	17889
Pentaflex wire	6760	11917
Control group	34126	49410
Experimental group	50789	76058

**Table 2:** Mechanical properties of the materials used in modeling

**Materials**	**Young's modulus (MPa)**	**Poisson's ratio**
Tooth	20300	0.26
Periodontal ligament (PDL)	0.667	0.49
Composite	16600	0.24
Spongy bone	13400	0.38
Cortical bone	34000	0.26
Pentaflex wire	90000	0.3

**Table 3:** Incisal and apical displacement of the mandibular anterior teeth in experimental and control groups (in microns)

**Models**		**E80**	**E90**	**E100**	**E110**	**C80**	**C90**	**C100**	**C110**	**E80-C80**	**E90-C90**	**E100-C100**	**E110-C110**
central	incisal	20.601	8.268	-5.394	-17.887	22.128	9.039	-0.155	-0.289	-1.527	-0.771	-5.239	-17.592
incisor	apical	0.12	-0.0234	-0.155	-0.295	0.111	-0.0277	-5.361	-18.703	0.009	0.0043	5.206	18.408
lateral	incisal	4.869	1.479	-1.956	-5.332	4.881	1.373	-0.119	-0.207	-0.012	0.106	-1.84	-5.13
incisor	apical	0.0147	-0.0671	-0.12	-0.213	0.00573	-0.0712	-2.177	-5.662	0.00897	0.0041	2.057	5.449
canine	incisal	1.806	0.372	-1.08	-2.496	1.137	0.257	-0.0288	-0.06	0.669	0.115	-1.05	-2.44
	apical	-0.079	-0.0955	-0.0273	-0.0536	-0.0848	-0.0985	-0.89	-1.879	0.0058	0.003	0.863	1.825
E=Experimental group,
C=Control group, 80, 90,
100 and 110=Incisor mandibular
plane angles (IMPA) of 80°C, 90°C,
100°C and 110°C

**Table 4:** Von Mises stresses in the PDL of the mandibular anterior teeth in experimental and control group (in Pascals)

**Central**	**Cervical**	**592**	**397**	**260**	**471**	**620**	**398**	**260**	**484**	**-28**	**-1**	**0**	**-13**
incisor	apical	5.72	7.01	9.71	1.95	5.96	7.05	9.79	3.98	-0.24	-0.04	-0.08	-2.03
lateral	cervical	168	106	109	174	177	111	180	4078	-9	-5	-71	-3900
incisor	apical	3.62	4.52	7.21	4.02	239	6.86	5.21	572	1.23	-2.34	2	-568
canine	cervical	122	66.1	49.1	75	126	67.5	48.7	73.7	-4	-1.4	0.4	1.3
	apical	5.43	2.11	3	1.28	4.66	1.91	1.34	1.28	0.77	0.2	1.66	0
E=Experimental group,
C=Control group, 80, 90,
100 and 110=Incisor mandibular
plane angles (IMPA) of 80°C,
90°C, 100°C and 110°C

**Table 5:** Strain energy in the mandibular anterior teeth for experimental and control groups (in milliJoules)

**Models**	**Strain energy**	**E-C**
E80	0.005497	-0.001662
C80	0.007159	
E90	0.005394	-0.00006
C90	0.005454	
E100	0.005742	-0.000016
C100	0.005758	
E110	0.006096	-0.000005
C110	0.006101	
E=Experimental group,
C=Control group, 80,
90, 100 and 110=Incisor
mandibular plane angles
(IMPA) of 80°C, 90°C,
100°C and 110°C
